# Genomic information on multidrug-resistant livestock-associated methicillin-resistant *Staphylococcus aureus* ST398 isolated from a Brazilian patient with cystic fibrosis

**DOI:** 10.1590/0074-02760160342

**Published:** 2017-01

**Authors:** Danielle F Lima, Renata WF Cohen, Géssica A Rocha, Rodolpho M Albano, Elizabeth A Marques, Robson S Leão

**Affiliations:** 1 Universidade do Estado do Rio de Janeiro, Faculdade de Ciências Médicas, Departamento de Microbiologia, Imunologia e Parasitologia, Rio de Janeiro, RJ, Brasil; 2 Fundação Oswaldo Cruz-Fiocruz, Instituto Nacional de Saúde da Mulher, da Criança e do Adolescente Fernandes Figueira, Rio de Janeiro, RJ, Brasil; 3 Universidade do Estado do Rio de Janeiro, Departamento de Bioquímica, Instituto de Biologia Roberto Alcântara Gomes, Rio de Janeiro, RJ, Brasil

**Keywords:** cystic fibrosis, Staphylococcus aureus, MRSA, ST398

## Abstract

Alarmingly, the isolation of methicillin-resistant *Staphylococcus aureus* (MRSA) has been increasing among patients with cystic fibrosis (CF). During a previous molecular characterisation of MRSA isolates obtained from patients with CF from Rio de Janeiro, Brazil, one isolate was identified as the ST398 clone, a livestock-associated (LA) MRSA. In this study, we report the draft genome sequence of an LA-MRSA ST398 clone isolated from a patient with CF.

The prevalence of respiratory tract methicillin-resistant *Staphylococcus aureus* (MRSA) in US patients with cystic fibrosis (CF) increased from 2% to 26% between 2000 and 2014 (CFF 2015). Epidemic MRSA clones have been found widespread in several regions around the world. Some of these clones were also found among patients with CF, including the Southern Germany (Molina et al. 2008), USA300, UK-EMRSA-3, and USA800/paediatric clones (Cocchi et al. 2011, Lima et al. 2014).

Until recently, there were no reports of another clone, ST398, which belongs to clonal complex (CC) 398, in patients with CF (Lima et al. 2014). This clone, known as livestock-associated methicillin-resistant *S. aureus* (LA-MRSA), first emerged among animals; however, it has already caused humans infections, such as folliculitis, osteomyelitis, endocarditis, and skin and soft tissue infections (van Belkum et al. 2008, Grisold et al. 2010, Schijffelen et al. 2010). Moreover, this LA-MRSA clone is widespread in Europe, Asia, and North America (Schijffelen et al. 2010, McCarthy et al. 2012), thus posing a threat to inpatients in some European hospitals, owing to its repertoire of resistance genes. In the present study, the genome of LA-MRSA ST398 was sequenced for the first time in South America.

A female patient with CF had been chronically colonised with *Pseudomonas aeruginosa* since she was four years old, and the only MRSA found was the ST398 clone when she was 17 years old. She lives in an urban community; however, in 2010, she spent time on a farm where she had recreational contact with farm animals. In the same month, after returning from her holiday, she suffered a respiratory exacerbation that was treated with intravenous antibiotics. During a previous molecular characterisation of MRSA isolates, one was identified as an ST398 clone obtained from her sputum sample (Lima et al. 2014).

This strain (MRSA 10152) was submitted for an antimicrobial susceptibility test by disk diffusion (cotrimoxazole, ciprofloxacin, erythromycin, clindamycin, gentamicin, rifampicin, tetracycline, and chloramphenicol). Vancomycin susceptibility was determined using broth microdilution and by molecular typing of staphylococcal cassette chromosome *mec* (SCC*mec*) and Panton-Valentine leucocidin (PVL) genes (Lima et al. 2014).

Whole genome sequencing of MRSA 10152 ST398 was performed using Illumina (Illumina Inc, USA) technology on a MiSeq instrument. A library was constructed with the Nextera XT DNA Library Preparation Kit (Illumina) and sequenced with a 500-cycle MiSeq Reagent kit v2 (Illumina). A total of 1,557,528 paired-end reads were obtained, which were corrected and assembled *de novo* by using the Spades 3.5 genome assembler (Bankevich et al. 2012). Contigs were submitted to the Rapid Annotation using Subsystem Technology (RAST) v.2.0 server (http://rast.nmpdr.org) and the subsystems were evaluated focusing on the presence of genes encoding antibiotic resistance, virulence mechanisms, biofilm production, adhesins, and host immune evasion. The results revealed a genome size of 2,878,277 bp and 24 RNA genes. Of the 2,726 identified coding sequences, 51% were included in 399 subsystems by RAST. Other databases, including ResFinder v.2.1 (https://cge.cbs.dtu.dk//services/ResFinder/), spaTyper v.1.0 (https://cge.cbs.dtu.dk/services/spatyper/), PHAge Search Tool (PHAST) (http://phast.wishartlab.com/) and BLASTP (GenBank), were used for more detailed genome annotation.

The MRSA 10152 ST398 isolate presented as *spa* type t034. This isolate’s resistance to erythromycin and a lincosamide (clindamycin) was associated with the presence of *ermC* and *lnuB* genes*,* respectively*,* while resistance to tetracycline was due to *tetM* and *tetK* genes. McCarthy et al. (2012) suggested the presence of the *tetM* gene as a possible marker to differentiate CC398 isolated from humans in contact with farm animals and those isolated from humans in the community. The presence of the *aadA2* gene confers resistance to aminoglycosides (streptomycin and spectinomycin). Resistance to fluoroquinolone (ciprofloxacin) is associated with mutations found in the *gyrA* and *grlA* genes.

A Tn916 conjugative transposon associated with the *ard*A gene (anti-restriction) was also found. This gene is related to the ability to acquire resistance genes associated with mobile genetic elements (McMahon et al. 2009). Two intact phages (PHAGE StaphyStauST398-3 and PHAGE StaphyStauST398-2) were also found.

The annotation results showed the presence of genes associated with intercellular adhesion (*icaA*, *icaB* and *icaC*), genes responsible for the synthesis of polysaccharide intercellular adhesin, and the regulatory genes *icaR*, *rbf*, *SarA* and *SigB.* Microbial surface components recognising adhesive matrix molecules (MSCRAMMs), such as CnaB, FnbA, Ebps, SdrC and SdrE were observed, along with extracellular adherence proteins Eap, Emp and Efp.

Genes associated with the production of toxins delta-haemolysin (*hld*), beta-haemolysin (*hlb*), and gamma-haemolysin (*hlgA, hlgB* and *hlgC*) were also present. Ishii et al. (2014) suggested that the *hlg*B gene may play an important role in encoding a major cytotoxin in *S. aureus* associated with lung infections. The *sak*, *chp*, and *scn* genes, which are related to escape from the human immune response in ST398 (Schijffelen et al. 2010), were not present in our isolate.

The draft genome of MRSA 10152 ST398 will aid the development of a detailed genomic analysis that will enhance the understanding and elucidation of the genetic diversity of this clone. The intensification of our microbiological knowledge is crucial to evaluate future clinical impact. This whole genome shotgun project has been deposited at DDBJ/ENA/GenBank under the accession number LZQL00000000. The version described in this paper is version LZQL01000000.
